# The localization of septo-cutaneous perforators of free fibular flaps determines the postoperative accuracy of maxillofacial reconstructions and should therefore be included in virtual surgical planning procedures

**DOI:** 10.1007/s10006-025-01366-y

**Published:** 2025-03-25

**Authors:** Manuel Khajehalichalehshtari, Tatjana Khromov, Babak Panahi, Boris Schminke, Henning Schliephake, Phillipp Brockmeyer

**Affiliations:** 1 Department of Oral and Maxillofacial Surgery, University Medical Center Goettingen, Goettingen, Germany; 2 Department of Diagnostic and Interventional Radiology, University Medical Center Goettingen, Goettingen, Germany; 3 Department of Clinical Chemistry, University Medical Center Goettingen, Goettingen, Germany

**Keywords:** Virtual surgical planning (VSP), Maxillofacial reconstruction, CAD/CAM, Surgical guides, Fibula free bone graft, Septo-cutaneous perforator

## Abstract

**Purpose:**

To investigate whether deviations in the localization of the main septo-cutaneous perforator (SCP) in maxillofacial reconstruction with free fibula flaps (FFF) lead to inaccuracies in the reconstruction result with respect to virtual surgical planning (VSP) procedures.

**Methods:**

The consecutive VSP planning data of a total of 24 patients who either underwent resection of a bone-destructive malignancy or underwent maxillofacial reconstruction with FFF due to another osteodestructive lesion were retrospectively analyzed together with the postoperative computed tomography (CT) control data set and the preoperative computed tomographic angiograms (CTA). The deviations of the VSP from the actual position of the main SCP were quantified morphometrically to evaluate the impact on the reconstruction accuracy.

**Results:**

Significant differences in bone segment surfaces (*p* = 0.0006) and bone segment volumes (*p* = 0.0001) were observed between VSP and postoperative reconstruction results. A significant positive linear relationship was found between the distance of the SCP from the inferior margin of the lateral malleolus (*p* = 0.0362, *R*^2^ = 0.1844) and the deviation of the SCP from the center of the VSP (*p* = 0.0016, *R*^2^ = 0.3700), with increasing root mean square (RMS) values indicating a less accurate reconstruction result. The multimodal regression model showed that the deviation of the SCP from the center of the VSP significantly affected the accuracy of the reconstruction result (*p* = 0.0046, *R*^2^ = 0.3345).

**Conclusions:**

The data provide evidence that the integration of the main SCP into the VSP procedures improves the predictability and accuracy of postoperative reconstruction outcomes.

## Introduction

The destruction of the jawbone in the maxillofacial region has a substantial negative impact on patients' ability to chew, speak, and swallow, which in turn affects their health-related quality of life (HRQOL) [1]. A number of factors may be responsible for the localized bone destruction. Oral squamous cell carcinoma (OSCC) is a particularly prevalent form of head and neck tumor and represents one of the most common cancers worldwide [2]. In the course of OSCC, the invasion and destruction of the adjacent jawbone is observed in a range of 12% to 56% of cases [3–6]. Therefore, the identification of bone invasion in OSCC is of paramount importance in the formulation of an efficacious treatment plan, which may entail the resection of the affected bone segments. In addition to bone-destructive malignancies, other conditions such as osteoradionecrosis [7], medication-related osteonecrosis of the jaw (MRONJ) [8], or inflammatory osteomyelitis [9] are increasingly being identified, necessitating the resection of affected bony areas.

It is of the utmost importance that resected bone segments be reconstructed in a manner that is both functional and aesthetically pleasing. This can be achieved through the utilization of free bone grafts, with particular consideration given to those obtained from the fibula, scapula, or iliac crest [10]. In particular, the free fibula flap (FFF) has emerged as the preferred option for long segmental bone reconstruction due to its unique blood supply, which allows for osteotomy and shaping to fit the specific anatomy of the defect being reconstructed [11]. Furthermore, the presence of septocutaneous perforators (SCP) at the transition from the lower to the middle third of the fibula enables the additional excision of a vascularized skin island for the purpose of providing soft tissue coverage [10].

The prevailing methodology for maxillofacial bone reconstruction entails a virtual surgical planning (VSP) approach, which is predicated upon a preoperative three-dimensional (3D) computed tomography (CT) dataset [12]. The intraoperative implementation of this VSP is conducted with the use of surgical guides, which are produced through a computer-aided design and computer-aided manufacturing (CAD/CAM) procedure. These guides facilitate a guided surgical approach [12]. The aforementioned techniques have markedly enhanced the precision, functionality, and aesthetic outcomes of maxillofacial reconstructions in recent years [12–16]. Current innovative approaches are increasingly exploring augmented reality (AR) technologies for intraoperative navigation and preoperative imaging techniques such as computed tomography angiography (CTA) and indocyanine green angiography to accurately assess vascular anatomy [17–19]. AR enables real-time visualization of anatomical structures for a more intuitive understanding of the surgical field and could therefore be an interesting virtual approach in contrast to physical surgical guides [17].

It is of the utmost importance to ensure that the optimal vascular pedicle length is maintained in order to guarantee the successful microsurgical anastomosis and healing of the FFF [20]. Accordingly, the bone segments to be harvested are typically planned in the VSP at a fixed distance of 7–8 cm from the inferior margin of the lateral malleolus in order to ensure the longest possible pedicle length [12]. However, the position of the SCPs, which are essential for harvesting the skin island, frequently deviates considerably from the intended position and has not yet been incorporated into the VSP [21].

**Fig. 1:**
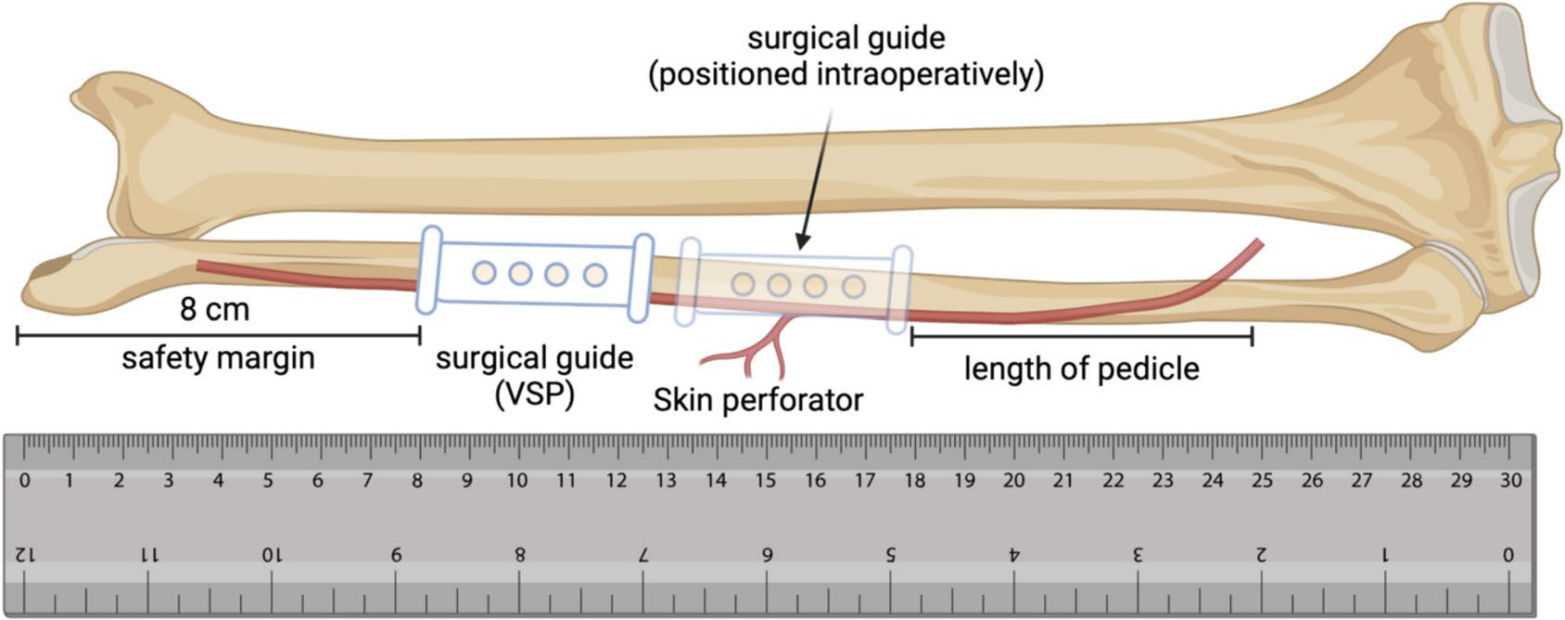
Research hypothesis: A greater deviation of the position of the main septo-cutaneous perforator (SCP) from the virtual surgical planning (VSP) leads to a greater deviation between planned and reconstructed bone segments and consequently to a less accurate reconstruction result

In this study, we conducted a retrospective analysis of the VSP planning data of 24 consecutive patients who were indicated for maxillofacial bone reconstruction using FFF, in conjunction with the preoperative computed tomographic angiograms (CTA) of the lower legs. Subsequently, we quantified the discrepancies between the VSP and the actual position of the main SCP in order to assess the impact on the accuracy of the postoperative reconstruction result. The results of this study demonstrate that the localization of the main SCP has a substantial influence on the postoperative reconstruction outcome. This may be attributed to discrepancies in the intraoperative positioning of the surgical guide (Fig. 1). Therefore, SCP integration into the VSP procedure is recommended.

## Materials and methods

### Patients

A retrospective evaluation was conducted on the maxillofacial reconstructions of a cohort of 24 patients diagnosed with an osteodestructive lesion (Table 1) and with the indication of maxillofacial bone resection and reconstruction. All patients underwent surgery in a consecutive series between May 2021 and April 2024. Primary bone reconstruction was performed in 22 cases and secondary reconstruction in 2 cases. The bone defect was in the mandible in 21 cases and in the maxilla in 3 cases. A commercially available VSP solution was employed, comprising CAD/CAM-fabricated surgical guides and titanium-made patient-specific implants (PSI) with a thickness of 2 mm. A morphometric analysis was conducted by a single trained examiner using preoperative VSP datasets, as well as preoperative and postoperative CT control datasets and CTA datasets. The following morphometric parameters were analyzed: the distance of the main SCP from the inferior margin of the lateral malleolus, the deviation of the main SCP from the center of the VSP, the bone surface and volume of the planned and reconstructed fibula bone segments, and the root mean square (RMS) of the superimposition of the VSP and the reconstructed bone segment. The study was approved by a clinical ethics committee (approval number 14/7/19).

**Table 1:** Baseline clinical characteristics and number of reconstruction segments

N	Osteodegenerative condition	Sex	Age	Number of reconstruction segments	Defect localization	Reconstruction modality	Radiation
1	Adenocarcinoma	Female	80	2	Mandible	Primary	No
2	Keratocyst	Male	70	2	Mandible	Primary	No
3	Ameloblastoma	Female	63	2	Mandible	Primary	No
4	Ameloblastoma	Male	15	2	Mandible	Primary	No
5	OSCC	Male	73	2	Mandible	Primary	No
6	Keratocyst	Male	34	2	Mandible	Primary	No
7	Fibrosarcoma	Male	51	2	Maxilla	Primary	Yes
8	OSCC	Male	85	2	Mandible	Primary	No
9	OSCC	Male	71	1	Mandible	Primary	No
10	Osteoradionecrosis	Male	74	2	Mandible	Secondary	Yes
11	Osteoradionecrosis	Female	68	2	Mandible	Primary	Yes
12	Osteoradionecrosis	Female	68	3	Mandible	Primary	Yes
13	OSCC	Female	58	3	Mandible	Primary	No
14	OSCC	Male	69	1	Mandible	Primary	No
15	Adenocarcinoma	Female	76	2	Maxilla	Primary	No
16	OSCC	Male	68	3	Mandible	Primary	No
17	OSCC	Female	76	2	Mandible	Primary	No
18	OSCC	Male	63	3	Mandible	Primary	No
19	MRONJ	Female	78	3	Mandible	Primary	Yes
20	Osteoradionecrosis	Male	55	3	Mandible	Primary	Yes
21	Osteoradionecrosis	Male	56	1	Mandible	Secondary	Yes
22	OSCC	Male	63	2	Mandible	Primary	No
23	OSCC	Female	51	2	Maxilla	Primary	No
24	OSCC	Female	61	2	Mandible	Primary	No

### Virtual surgical planning (VSP) solution

The VSP, surgical guide, and PSI fabrication were conducted by a certified commercial partner in accordance with the principles of CAD/CAM-based surgical procedures. For these purposes, the CT datasets of the head and neck region, as well as the CTA images of the lower extremities, were transmitted in encrypted form via the partner's online platform. The entire planning process, including the segmentation and creation of the 3D anatomical models as well as the virtual resection and subsequent modeling of the PSI and surgical guides, was conducted by a trained 3D designer from the commercial partner. Once the preliminary design was finalized, an online meeting was held with the surgeon to discuss and implement any suggested modifications. Following the surgeon's approval of the final design, the manufacturing process was initiated.

### Morphometric evaluation

**Fig. 2:**
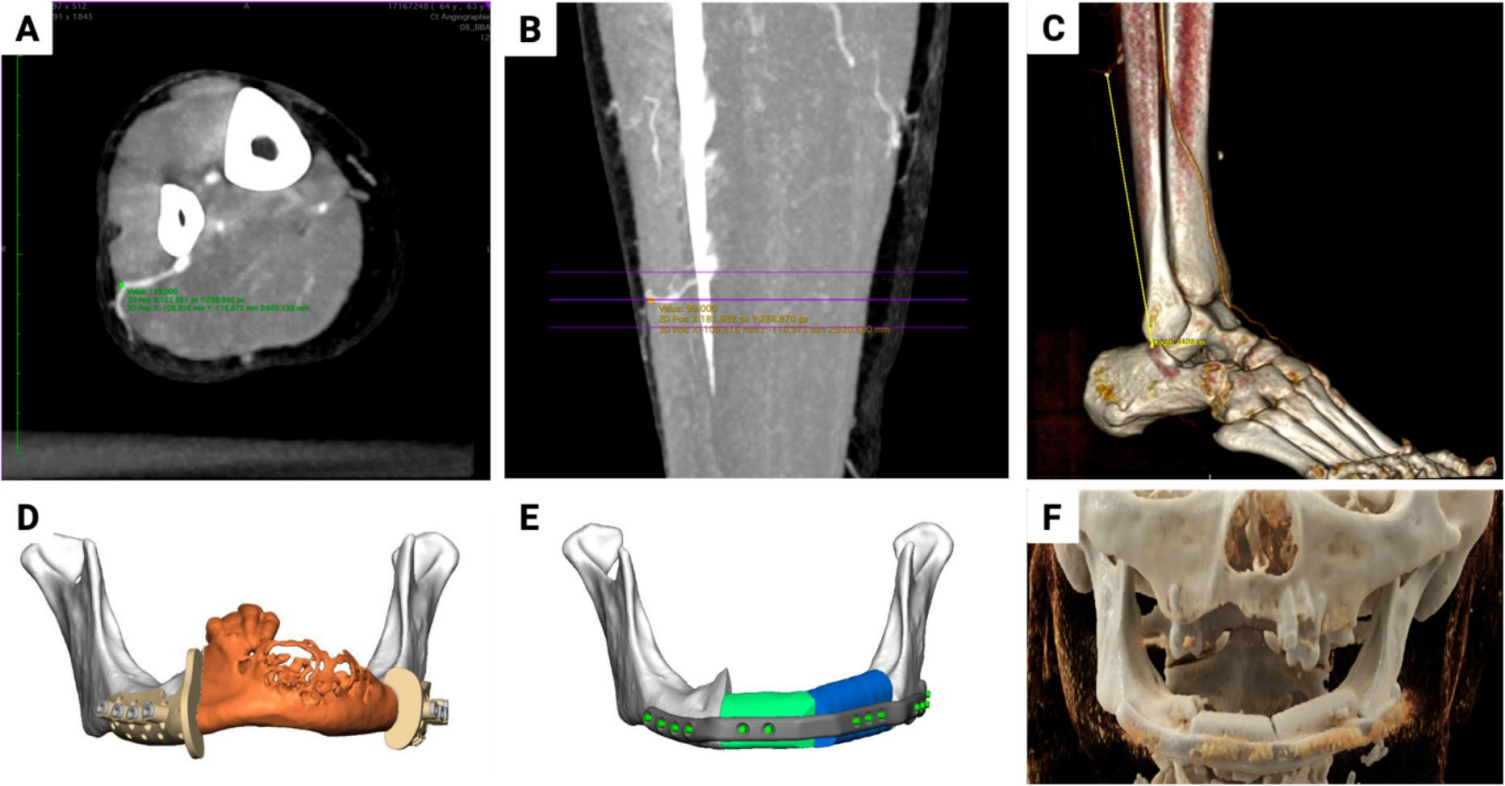
Example of mandibular reconstruction with FFF consisting of two bone segments. (**A**) Visualization of the SCP on CTA in the axial cross-sectional images. (**B**) Visualization of the SCP on CTA in the coronal cross-sectional images using MIP. (**C**) Visualization of the SCP in cinematic rendering. (**D**) Visualization of the VSP with the orange marked resection area with osteodestructive lesion and the virtually modeled surgical guides. (**E**) VSP showing the planned reconstruction with two bone segments and modeling of the PSI. (**F**) Postoperative control CT with cinematic rendering showing the reconstruction result

**Fig. 3:**
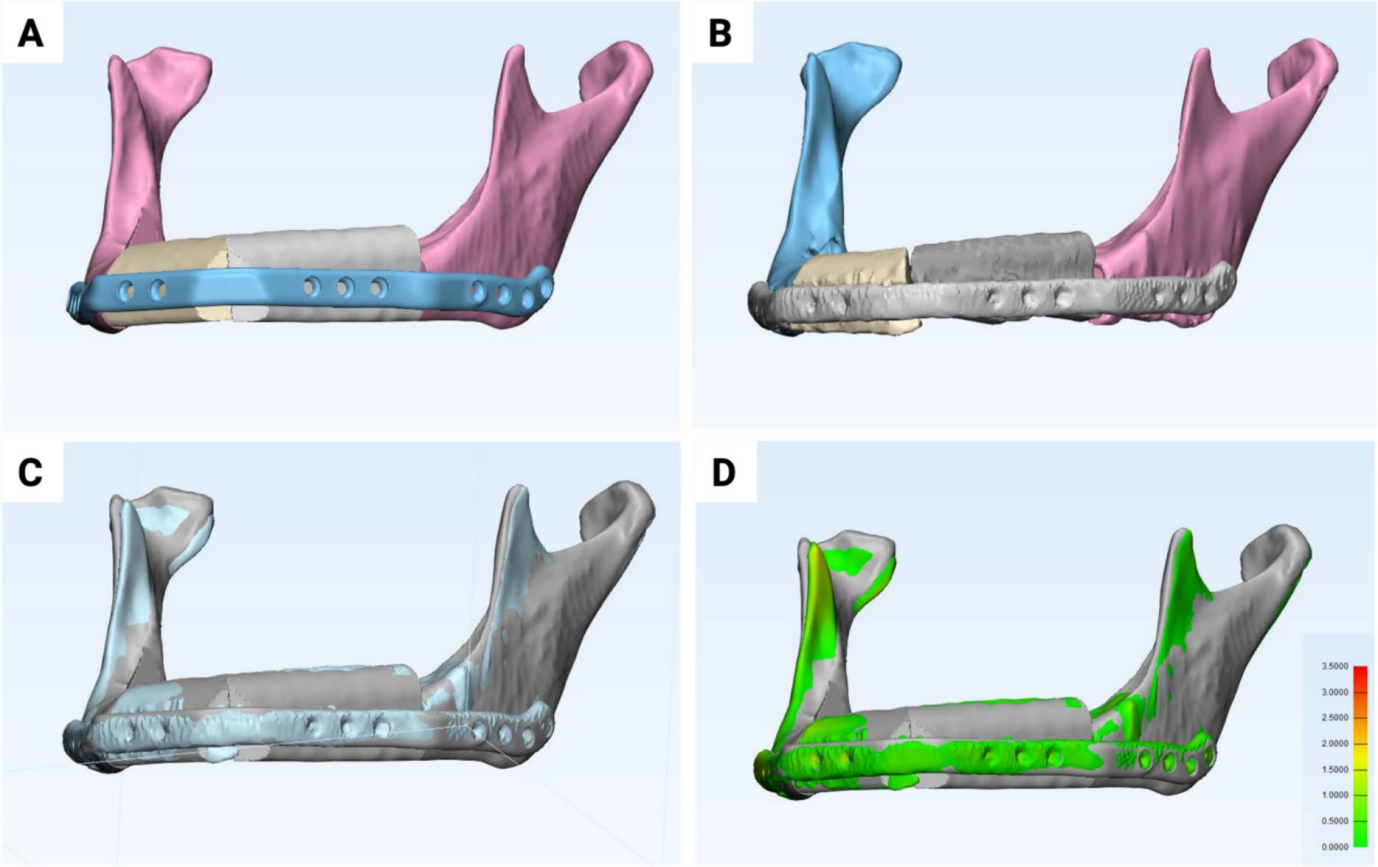
Illustration of morphometric evaluation of mandibular bone reconstruction accuracy. (**A**) 3D model of the planned reconstruction in the VSP. (**B**) 3D model of the postoperative reconstruction result. (**C**) Superimposition of the postoperative reconstruction result with the VSP. (**D**) Visualization of the deviations using a heat map showing the RMS values between 0 (green) and 3.5 (red)

**Fig. 4:**
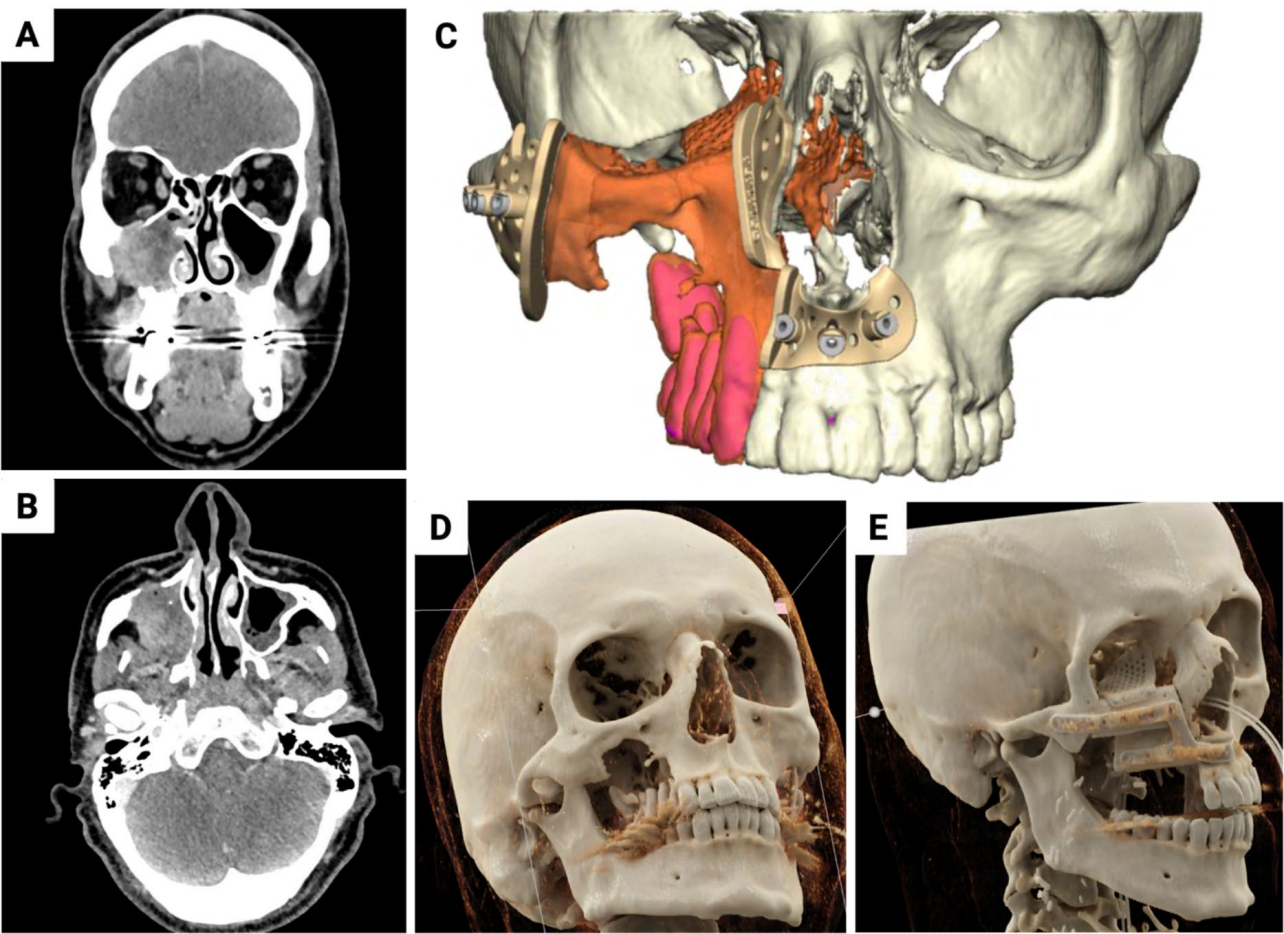
Illustration of an exemplary midface reconstruction with FFF consisting of two bone segments and an additional PSI for orbital floor reconstruction. (**A**) Illustration of the invasive fibrosarcoma in the right maxillary sinus in coronal cross-sectional imaging. (**B**) Axial cross-sectional view of the tumor. (**C**) VSP with planned resection (orange and red) and modeling of the surgical guides. (**D**) Cinematic rendering of the CT image of the initial findings with osteolytic process of the right maxillary sinus. (**E**) Postoperative control CT, cinematic rendering showing the reconstruction result

**Fig. 5:**
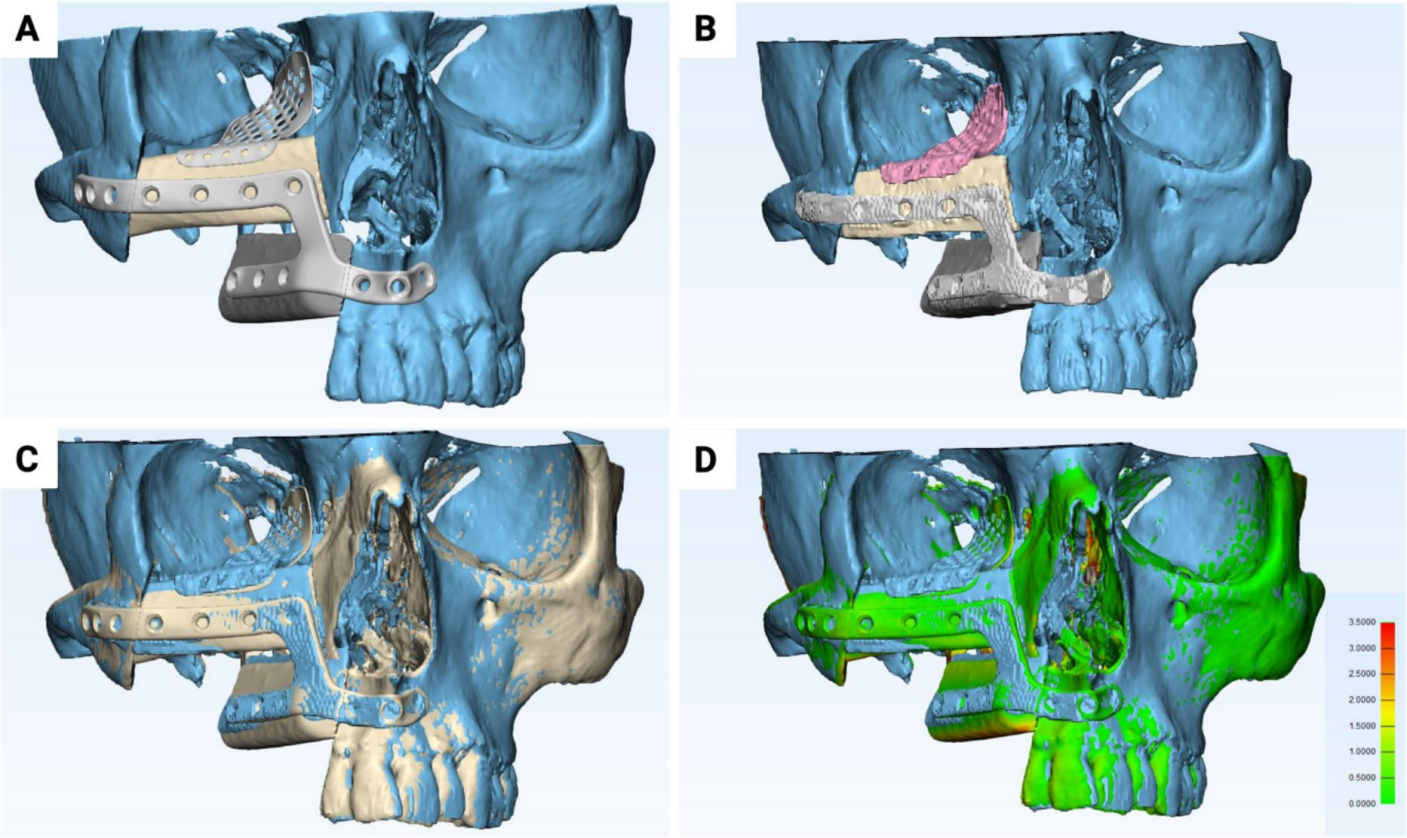
Illustration of morphometric evaluation of midface bone reconstruction accuracy. (**A**) 3D model of the planned reconstruction in the VSP. (**B**) 3D model of the postoperative reconstruction result. (**C**) Superimposition of the postoperative reconstruction result with the VSP. (**D**) Visualization of the deviations using a heat map showing the RMS values between 0 (green) and 3.5 (red)

The preoperative CTA data (foot to knee) with a slice thickness of 1 mm was utilized to quantify the distance of the main SCP, at the transition of the lower to the middle third of the fibula, from the inferior margin of the lateral malleolus. In this context, the open-source Horos software (version 3.3.6, LGPL license, Horosporject.org) was employed in conjunction with maximum intensity projection (MIP, Figs. 2A, B, and C). The preoperative VSP data were provided by the commercial partner (Figs. 2D, E, 3, and 4C), and the postoperative control datasets were segmented using Mimics Innovation Suite software (version 3.0, Materialise, Leuven, Belgium) to create 3D virtual anatomical models (Figs. 3 and 5). The 3-matic software (version 17.0, Materialise, Leuven, Belgium) was employed to quantify the surface area and volume of the fibula bone segments and to superimpose the VSP with the outcome of the complete bone reconstruction via the part-comparison algorithm (Figs. 3D and 5D). The methodology allowed for the evaluation of discrepancies, as indicated by the RMS values.

### Statistical analysis

Continuous data were expressed as minimum, mean, and maximum values with standard deviation and 95% confidence interval. A series of tests, including the Kolmogorov–Smirnov, Shapiro–Wilk, and Anderson–Darling tests, were performed to determine the normal distribution of all data sets. Comparative analysis of bone segment volume and surface was performed using the Wilcoxon rank-sum test (two-tailed) to determine significant differences between VSP and reconstruction results. To test the correlation between abnormal SCP localization and increasing RMS values indicating an inaccurate reconstruction result, first simple linear regression analyses and then a multiple regression model were performed. The quality of the model fit and the fulfillment of the model assumptions (including linearity, error independence, error homoscedasticity, normality, and no multicollinearity) were checked by careful inspection of the residual plots. The quality of the model fit was assessed by the coefficient of determination R^2^. The t-test was used to check whether the calculated slope was significantly different from zero. All statistical analyses were performed with a significance level of α = 5% using Prism software (version 9.5, GraphPad, La Jolla, CA, USA). A p-value less than 0.05 was considered statistically significant.

## Results

**Fig. 6:**
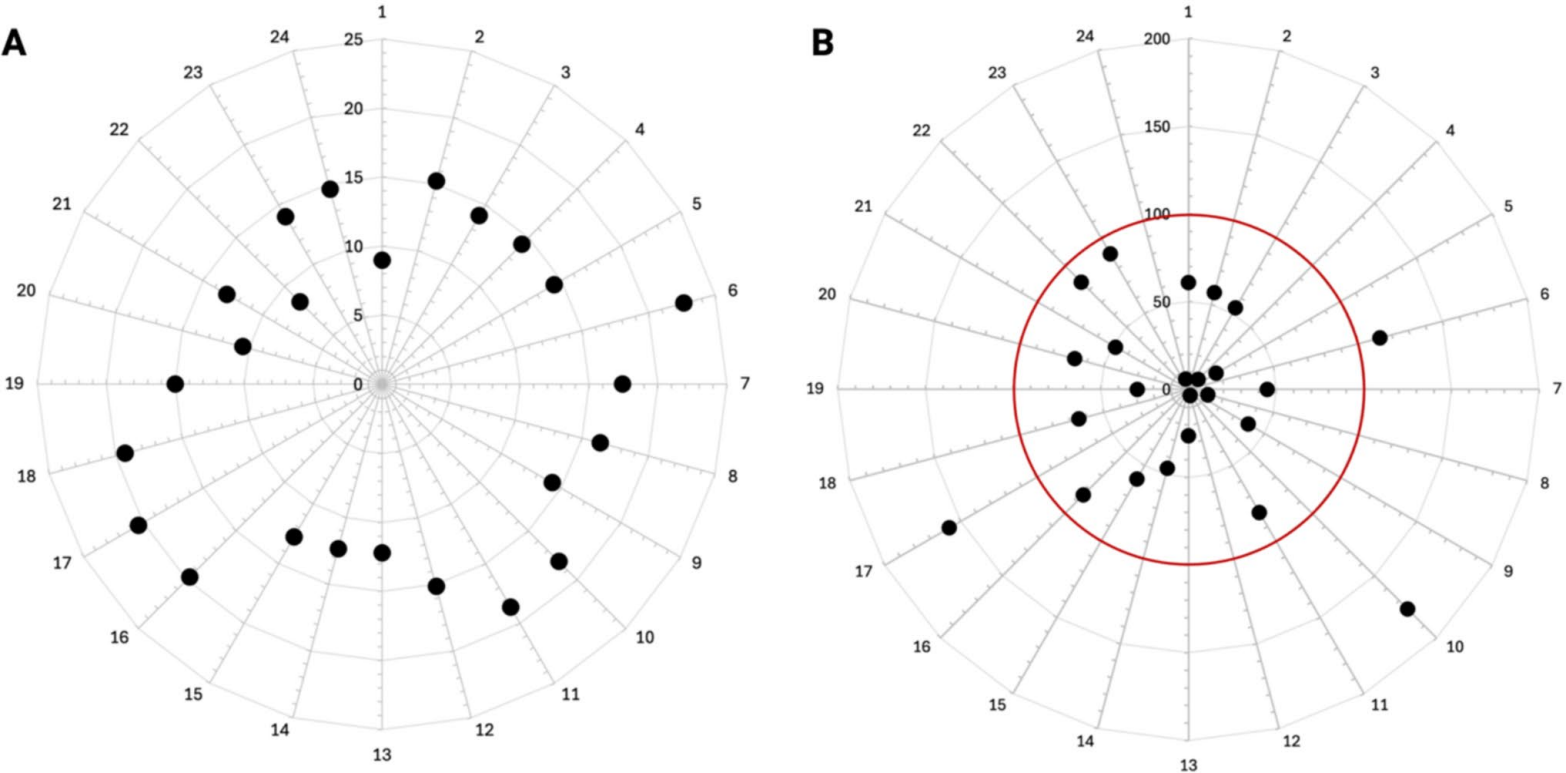
(**A**) Distribution of the main SCP from the inferior margin of the lateral maleolus in cm in all cases. (**B**) Percentage deviation of the main SCP from the center of the VSP in all cases

**Fig. 7:**
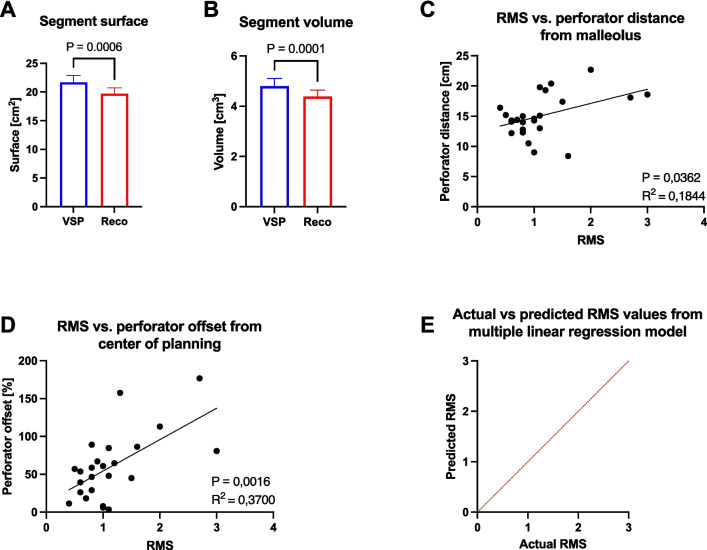
(**A**) Comparison of segmental surfaces between VSP and postoperative reconstruction results. *P* value from Wilcoxon matched-pairs signed rank test, *n* = 51. (**B**) Comparison of segmental volumes between VSP and postoperative reconstruction result. *P* value from Wilcoxon matched-pairs signed rank test, *n* = 51. (**C**) Relationship between root mean square (RMS) and skin perforator distance from the inferior margin of the lateral maleolus. *P* value and R2 value from simple linear regression analysis, *n* = 24. (**D**) Relationship between RMS and percentage deviation of the skin perforator from the center of the VSP. *P* value and R2 value from simple linear regression analysis, *n* = 24. (**E**) Presentation of the results of the multiple regression analysis model using the actual vs. predicted scatter plot, *n* = 24

The clinical baseline characteristics of the patient collective, as well as a summary of the morphometric analysis data, are presented in Tables 1 and 2. In summary, the morphometric evaluation revealed a considerable range of SCP localization, with instances occurring at the inferior margin of the lateral malleolus at varying distances (from 8.40 cm to 22.70 cm, as illustrated in Fig. 6A). This resulted in a range of SCP deviations, varying from 3.60% to 176.80% (as illustrated in Fig. 6B), from the center of the VSP. Moreover, the analysis demonstrated a statistically significant reduction in bone segment surfaces (*p* < 0.0001, Fig. 7A) and bone segment volumes (*p* < 0.0001, Fig. 7B) in the postoperative reconstruction result in comparison to the VSP. The deviation analysis, conducted by superimposing the VSP and the postoperative reconstruction results, revealed RMS values between 0.40 and 3.00, indicating disparate levels of postoperative reconstruction accuracy. The results of the simple linear regression analysis indicated a positive correlation between the distance of the main SCP from the inferior margin of the lateral malleolus and increasing RMS values (indicating less accurate reconstruction results; *p* = 0.0362, *R*^2^ = 0.1844, Fig. 7C). Moreover, a notable positive correlation was identified between the increasing deviations of the primary SCP from the center of the VSP and the increasing RMS values (*p* = 0.0016, *R*^2^ = 0.3700, Fig. 7D). The final multiple linear regression model confirmed that the deviation of the SCP from the center of the VSP significantly influenced the RMS values (*p* = 0.0046, *R*^2^ = 0.3345, Table 3), thereby affecting the postoperative reconstruction result.

**Table 2:** Descriptive statistics of the morphometric evaluation

Parameter	Min	Mean	Max	SD	95% CI
Surgical guide length [cm]	5.30	11.00	15.90	3.35	9.58, 12.41
Planning distance from the inferior margin of the lateral maleolus [cm]	7.00	7.79	9.50	0.64	7.52, 8.06
Main SCP distance from the inferior margin of the lateral maleolus [cm]	8.40	15.08	22.70	3.53	13.59, 16.57
Main SCP offset from center of planning [%]	3.60	59.67	176.80	44.07	41.06, 78.28
Bone segment surface VSP [cm^2^]	9.90	21.71	46.70	8.39	19.35, 24.07
Bone segment surface reco [cm^2^]	8.30	19.69	37.00	7.55	17.56, 21.81
Bone segment volume VSP [cm^3^]	1.10	4.802	10.10	2.12	4.21, 5.40
Bone segment volume Reco [cm^3^]	1.00	4.38	9.20	1.87	3.86, 4.91
Root mean square (RMS)	0.40	1.13	3.00	0.65	0.86, 1.40

**Table 3:** Results of the multiple linear regression model with RMS as the outcome variable

Variable	95% CI	SE	*R*^2^	*P*
Intercept	−2.805, 5.369	1.9545		0.5182
Surgical guide length [cm]	−0.0449, 0.1811	0.0538	0.6558	0.2215
Planning distance from the inferior margin of the lateral maleolus [cm]	−0.6028, 0.2731	0.2085	0.3759	0.4393
Main SCP distance from the inferior margin of the lateral maleolus [cm]	−0.0415, 0.1066	0.0353	0.2816	0.3678
Number of reconstruction segments	−0.8110, 0.1873	0.2376	0.5131	0.2058
Main SCP offset from center of planning [%]	0.0033, 0.015	0.0029	**0.3345**	**0.0046**

## Discussion

The progression of osteodestructive disease and the ablative surgical treatment of destroyed sections of the bone of the maxillofacial region are both associated with considerable functional and aesthetic restrictions for patients, which have a negative impact on their HRQOL [1]. It is thus evident that the reconstruction of lost bone sections represents a pivotal aspect of the treatment plan, with the objective of restoring both function and aesthetics [22]. A multitude of surgical techniques and reconstruction procedures, including the utilization of fibula, scapula, or iliac crest bone grafts, serve as the foundation for the reconstruction of these defects [10].

Previously, bone reconstruction in the maxillofacial region was conducted via a freehand procedure, which necessitated a significant investment of time and was markedly influenced by the surgical expertise of the operating team [23]. Nevertheless, a preoperative VSP with subsequent fabrication of surgical guides using CAD/CAM technologies has now become a standard procedure for a guided surgical approach [24]. These techniques have allowed for a more precise and personalized surgery, accurate flap positioning, reduced operative and graft ischemia times, and improved postoperative aesthetic outcomes [25–27]. Although AR-assisted intraoperative navigation and virtual overlay of VSP on anatomical structures have the potential to replace physical guides in the future, AR tracking is still error prone, and real-time processing of large amounts of data is computationally intensive and requires improved hardware and software solutions, making this innovative technology clinically inaccessible to date [17].

The FFF has become the gold standard for maxillofacial reconstructions, particularly in the context of mandibular bone defects [10, 28]. The suitability of the bone for modelling the desired shape through intermediate osteotomies is enhanced by its secure vascular supply, robust bone structure, and extended vascular pedicle [29]. The bone segments may be stabilized for in situ healing through the use of either pre-bent prefabricated osteosynthesis plates or PSIs [12]. Moreover, the FFF has been demonstrated to confer several advantages over the scapular graft, including more precise realization of the VSP and a more aesthetically pleasing postoperative result [25]. Furthermore, the FFF, which encompasses SCPs, offers the option of transplanting a vascularized skin island, which can be employed to cover a non-voluminous soft tissue defect [29].

SCP localization is not yet part of the defined VSP standard, and there is a remarkable range of SCP localization along the lower extremity [21, 30]. Although there are limitations in the retrospective study design, the small number of cases that can be however considered a meaningful population compared to others [31–33], and the morphometric analysis by a single observer, this study provides the first indication that a deviating SCP position may significantly affect the postoperative reconstruction outcome.

Increased RMS values, indicating poorer reconstruction fit, were significantly correlated with a large distance of the SCP from the inferior margin of the lateral malleolus and with a large deviation of the SCP from the center of the VSP. While the results of the linear regression analysis for the distance of the SCP from the inferior margin of the lateral malleolus must be interpreted with caution due to the low R^2^ value, the analysis for the deviation of the SCP from the center of the VSP showed a relevant correlation, which was also confirmed by the multiple regression model.

We hypothesize that due to a discrepancy in the identified position of the SCP, the surgical guide must be positioned more proximally to the fibula than originally planned in the VSP (Fig. 1). This misalignment of the surgical guide results in an inadequate fit in this area due to the altered bone morphology, as well as a change in the segmental bone surface and volume, leading to a less accurate postoperative reconstruction result compared to the VSP.

These results advocate the integration of SCP into the VSP for optimal reconstruction results. It is unfortunate that there is considerable inter-individual variability in the diameter of the SCPs, which presents a challenge in visualizing these small vessels in many cases. In this study, for instance, CTAs with a layer thickness of 1 mm were employed, which precluded the identification of vessels smaller than the visible area. Nevertheless, it has been demonstrated that CTA is a more suitable modality for mapping perforators than MRA [34], and is more readily available, adequately accurate, and cost-effective [35–37]. Future enhancements in imaging, such as a further reduction in layer thickness (e.g. 0.6 mm) and the adaptation of the contrast agent applied, must be employed to map detailed structures such as SCP localization during preoperative diagnostics and planning in order to integrate them into the VSP for optimal reconstruction results. The distance of the SCP to fixed anatomical structures, such as the inferior margin of the lateral malleolus, can then be transferred to the virtual fibula in the VSP and the bone segments adapted accordingly.

However, it is important to note that the bone segments to be harvested may move further proximal on the fibula, which can significantly reduce the vascular pedicle length. In most of the clinical cases presented here, the reconstructions were primary bone reconstructions of the mandible, where a shorter pedicle length is usually unproblematic for microvascular anastomosis and a sufficient transplant healing. However, it should be noted, that different clinical conditions, such as extensive maxillary/midface reconstructions or secondary reconstructions following previous surgery or radiotherapy, with compromised vascular supply, can require the fully available vascular pedicle length, making it is necessary to deviate from the proposed procedure.

## Conclusions

Deviations in the localization of the main septo-cutaneous perforator from the virtual surgical planning during free fibula flap harvesting can result in inaccuracies in the reconstruction of maxillofacial bone defects. It is therefore recommended that the perforator be visualized preoperatively and integrated into virtual surgical planning procedures to ensure optimal outcomes.

## Data Availability

No datasets were generated or analysed during the current study.
